# MyelTracer: A Semi-Automated Software for Myelin *g*-Ratio Quantification

**DOI:** 10.1523/ENEURO.0558-20.2021

**Published:** 2021-07-21

**Authors:** Tobias Kaiser, Harrison Mitchell Allen, Ohyoon Kwon, Boaz Barak, Jing Wang, Zhigang He, Minqing Jiang, Guoping Feng

**Affiliations:** 1 McGovern Institute for Brain Research, Cambridge, MA 02139; 2 Department of Brain and Cognitive Sciences, Massachusetts Institute of Technology, Cambridge, MA 02139; 3 Department of Electrical Engineering and Computer Science, Massachusetts Institute of Technology, Cambridge, MA 02139; 4 The Sagol School of Neuroscience and The School of Psychological Sciences, Tel Aviv University, Tel Aviv, 6997801, Israel; 5 F.M. Kirby Neurobiology Center, Boston Children’s Hospital, and Department of Neurology and Ophthalmology, Harvard Medical School, Boston, MA 02115; 6 Stanley Center for Psychiatric Research, Broad Institute of MIT and Harvard, Cambridge, MA 02142

**Keywords:** electron microscopy, *g*-ratio, myelin, toolbox

## Abstract

In the central and peripheral nervous systems, the myelin sheath promotes neuronal signal transduction. The thickness of the myelin sheath changes during development and in disease conditions like multiple sclerosis. Such changes are routinely detected using electron microscopy through *g*-ratio quantification. While *g*-ratio is one of the most critical measurements in myelin studies, a major drawback is that *g*-ratio quantification is extremely laborious and time-consuming. Here, we report the development and validation of MyelTracer, an installable, stand-alone software for semi-automated *g*-ratio quantification based on the Open Computer Vision Library (OpenCV). Compared with manual *g*-ratio quantification, using MyelTracer produces consistent results across multiple tissues and animal ages, as well as in remyelination after optic nerve crush, and reduces total quantification time by 40–60%. With *g*-ratio measurements via MyelTracer, a known hypomyelination phenotype can be detected in a Williams syndrome mouse model. MyelTracer is easy to use and freely available for Windows and Mac OS X (https://github.com/HarrisonAllen/MyelTracer).

## Visual Abstract

**Figure:**
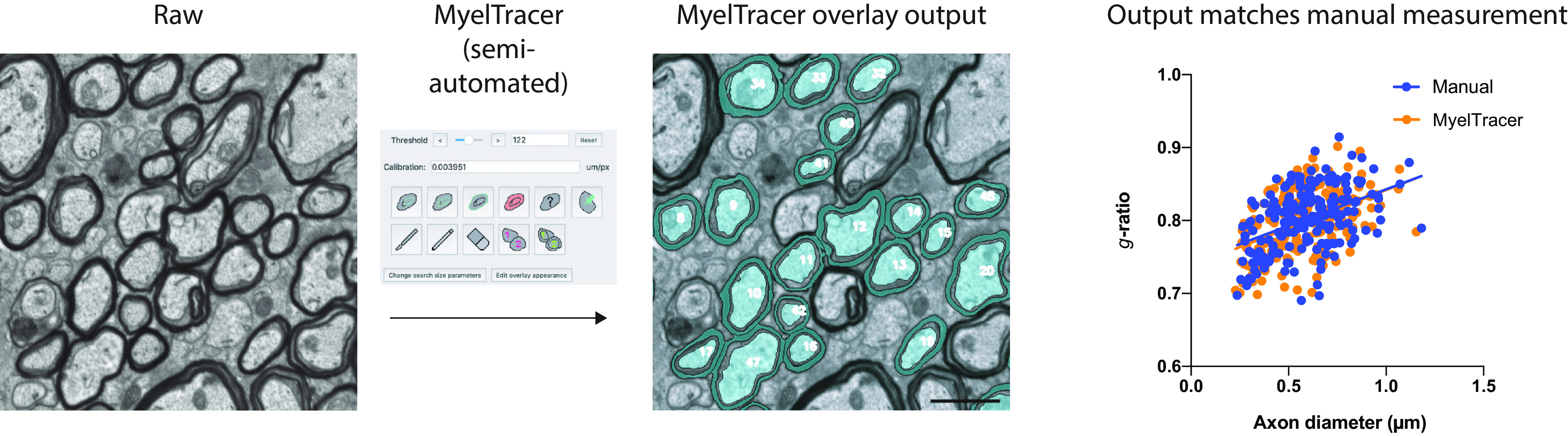

## Significance Statement

An easy-to-use software suite for *g*-ratio quantification in myelin ultrastructure micrographs is currently unavailable, but it is much needed to streamline the study of myelin in homeostasis and disease-related conditions. We used computer vision libraries to develop a freely available software to facilitate studies of myelination.

## Introduction

The myelin sheath is essential for proper neuronal functions in central and peripheral nervous systems. Oligodendrocytes and Schwann cells produce the myelin sheath through lamellar enwrapping of axons (Simons and Trotter, 2007; Salzer, 2015; Hughes and Appel, 2016; Stadelmann et al., 2019). The myelin sheath insulates axons, thereby preserving axonal integrity and promoting neuronal signal transduction (Sherman and Brophy, 2005; Nave, 2010). Under physiological conditions, changes in myelin contribute to behaviorally relevant neural plasticity mechanisms and experience-dependent sensory adaptations (Gibson et al., 2014; McKenzie et al., 2014; Hughes et al., 2018).

Abnormal changes in myelin are prominent features of many clinical pathologies. Ultrastructural myelin abnormalities, such as hypomyelination, myelin degeneration, and tomaculi, are cardinal features of prototypic white-matter diseases like multiple sclerosis, Pelizaeus Merzbacher disease, and Charcot-Marie-Tooth disease (Krajewski et al., 2000; Sander et al., 2000; Franklin and ffrench-Constant, 2008; Lin and Popko, 2009; Duncan and Radcliff, 2016). In addition, myelin abnormalities have recently been discovered as common features of neurodevelopmental disorders, including Pitt–Hopkins syndrome, Rett syndrome, autism spectrum disorders (ASDs), and William syndrome (Zhao et al., 2018; Barak et al., 2019; Phan et al., 2020).

In both preclinical and clinical settings, pathologic myelin abnormalities are often identified by studying myelin ultrastructure. Specifically, researchers use *g*-ratio as a metric for the relative thickness of the myelin sheath in cross-sectional micrographs (Rushton, 1951). Since manual myelin tracing is a major bottleneck in *g*-ratio quantification, tools that streamline this process will tremendously facilitate future studies investigating activity-dependent homeostatic and pathologic changes to the myelin sheath.

Several non-automated, semi-automated, and fully automated toolboxes are currently available. They include G-ratio for ImageJ, AxonSeg, and AxonDeepSeg (Goebbels et al., 2010; More et al., 2011; Bégin et al., 2014; Zaimi et al., 2016, 2018; Janjic et al., 2019). Collectively, these tools have facilitated different quantitative measurements of myelin ultrastructure, such as the number of axons and *g*-ratio. However, the current tools with more automation impose difficult requirements on their users, such as preprocessing data or modifying parameters by writing code. These drawbacks have impeded the tools’ widespread adoption by the scientific community. We identified four major requirements of an open access toolbox that would significantly facilitate myelin analyses: (1) an intuitive graphical user interface that can be installed and used without running code; (2) consistent quantification results compared with manual analysis; (3) significantly less time-consuming; and (4) well-organized output data files and overlay images for publication and *post hoc* quality control.

Here, we report the development and validation of MyelTracer, an easy-to-use software that fulfills these critical requirements. Built using OpenCV and PyQt5’s GUI toolkit, MyelTracer replaces the manual tracing of axons and myelin with semi-automated, threshold-dependent outlining. Compared with manual quantification, semi-automated quantification of axon diameters and *g*-ratio via MyelTracer produces consistent results across several tissues and developmental time points while reducing total quantification time by 40–60% depending on the region. Furthermore, using *g*-ratio measured with MyelTracer, we can detect a known hypomyelination phenotype in a Williams syndrome mouse model, as well as in remyelination after optic nerve crush, indicating the applicability of MyelTracer for studies in disease contexts. To ensure MyelTracer’s all-in-one output functionality, we included a manual tracing function and counters for quantification of abnormally shaped sheaths and fraction of myelinated axons, respectively. MyelTracer is a valuable tool for the study of myelin ultrastructure and is freely available to the public as an installable software for Mac and PC (https://github.com/HarrisonAllen/MyelTracer).

## Materials and Methods

### Animal work

All animal procedures were performed in accordance with the Massachusetts Institute of Technology animal care committee’s regulations.

### Code accessibility

The code/software described in the paper is freely available online at https://github.com/HarrisonAllen/MyelTracer. The code is available as Extended Data 1.

10.1523/ENEURO.0558-20.2021.ed1 Extended Data 1 We recommend downloading MyelTracer directly from the GitHub repository, at [GitHub link, blinded for review]. Download Extended Data 1 (containing source code). Download Extended Data 1, ZIP file.

### Software development

Outline extraction of axons is performed using an implementation of OpenCV in Python. The processing is done as follows. First, the color space of the image is converted from RGB to grayscale, so that all pixels have a scalar magnitude between 0 and 255.

cvtColor(src, COLOR_BGR2GRAY)

Then, a bilateral filter is applied to the source image to reduce noise and smooth edges.

bilateralFilter(src, d = 9, sigmaColor = 75, sigmaSpace = 75)

A binary thresholding filter converts pixel values below the user defined threshold, *t*, to black, and those above *t* to white.

threshold(src, thresh=t, maxval = 255, type=THRESH_BINARY)

Lines drawn by the user are then applied to the thresholded image using the *polylines* function.

Contours are extracted as the boundaries between the black and white pixels of the thresholded image.

findContours(src, mode=RETR_TREE, method=CHAIN_APPROX_SIMPLE)

Contours with fewer than five vertices are filtered out using the *len* function as well as contours that do not fall within the user-defined area constraints, *Min Size* and *Max Size*, calculated using the *contourArea* function.

Once selected, contours for each feature, axon, inner myelin sheath, and outer myelin sheath, are grouped together by checking whether the features nest within one another using *pointPolygonTest*.

Exported data are written in a plain-text csv document for compatibility with most spreadsheet softwares. The exported features are organized by the order in which the user selected the axon feature. Area is calculated using *contourArea*, perimeter is calculated using *arcLength*, diameter is calculated using $2\cdot \sqrt{area/\pi }$, and *g*-ratio is calculated using $\sqrt{innerMyelinArea/OuterMyelinArea.}$

The extraction software is wrapped in a custom-designed *PyQt5* GUI, and both Windows and MacOS installers are generated using the *fman Build System* (fbs). MyelTracer has been tested and proven to work on Windows 10 and MacOS Catalina 10.15.

### Electron microscopy and *g*-ratio tracing

#### Sample preparation and imaging

One- and two-month-old male and female C57BL/6J mice were deeply anesthetized with isoflurane and transcardially perfused with 30 ml ice-cold saline solution followed by 30-ml fresh ice-cold 2.5% glutaraldehyde + 2% PFA in 0.1 m sodium cacodylate buffer (pH 7.4). Brains, optic nerves and sciatic nerves were dissected and kept in the fixation solution overnight at 4°C. Small pieces (1- to 2-mm cubes) of tissue samples from the dorsal corpus callosum at the level of the fornix (bregma −0.94 mm), optic nerve, and sciatic nerve were postfixed for at least 2 h at room temperature in the fixation solution. They were then washed in 0.1 m sodium cacodylate buffer and postfixed with 1% osmium tetroxide/1.5% potassium ferrocyanide for 1 h before being washed in water three times and incubated in 1% aqueous uranyl acetate for 1 h. This was followed by two washes in water and subsequent dehydration in grades of alcohol (10 min each; 50, 70, 90 and 2 × 10 min 100%). The samples were then put in propylene oxide for 1 h and infiltrated overnight in a 1:1 mixture of propylene oxide and epoxy resin mixture (TAAB Epon; Marivac Canada). The following day, the samples were embedded in TAAB Epon and polymerized at 60°C for 48 h. Ultrathin sections (∼60 nm) were cut on a Reichert Ultracut S microtome (Leica), picked up on copper grids stained with lead citrate and examined in a JEOL 1200EX transmission electron microscope (JEOL USA). The images were recorded with a 2k CCD camera (Advanced Microscopy Techniques).

### Quantification of *g*-ratio

To compare manually quantified *g*-ratio and those quantified with MyelTracer, three images for each region of interest were analyzed manually and using MyelTracer. Each image was analyzed first with MyelTracer, which automatically numbered every quantified axon. For manual *g*-ratio quantification, the same axons were manually numbered and then traced using ImageJ. To compare the control and Gtf2i-knock-out group, raw images were obtained from Barak and coworkers (Barak et al., 2019), blinded of their genotypes and quantified using MyelTracer. To test MyelTracer’s ability to quantify *g*-ratios in remyelinating tissues, raw images of the optic nerve from control animals and animals 28 d post-injury with Montelukast and Pexidartinib treatment were obtained from Wang et al. (2020). For each experiment comparing manual tracing with MyelTracer, several micrographs were obtained from preparations of two to three mice per anatomic region. Within these micrographs, at least 100–300 axons were analyzed depending on the anatomic region. For testing MyelTracer’s capability to detect a hypomyelination phenotype in a Williams syndrome model, several micrographs from three mice per genotype with a total of 332 and 455 axons were analyzed for control and Gtf2i fl/fl; Nex-Cre mice, respectively. For optic nerve, sciatic nerve, and corpus callosum, three different researchers recorded image quantification times. Numbers denoting measured axons in representative images were enlarged for easier visualization. MyelTracer was run on a MacBook Pro (2.8 GHz, 16 GB RAM, Intel Iris Plus Graphics 655 1536 MB and macOS Catalina version 10.15.2.), Windows 10 (Intel Core i5-7300HQ CPU 2.50 GHz, 8GB RAM, NVIDIA GeForce GTX 1050 Ti), and MacBook Air (Intel Core i5 1.3 GHz CPU, 4GB RAM, Intel HD Graphics 5000, macOS Catalina version 10.15.2).

## Results

### MyelTracer is a semi-automated software using computer vision

**Figure 1.**
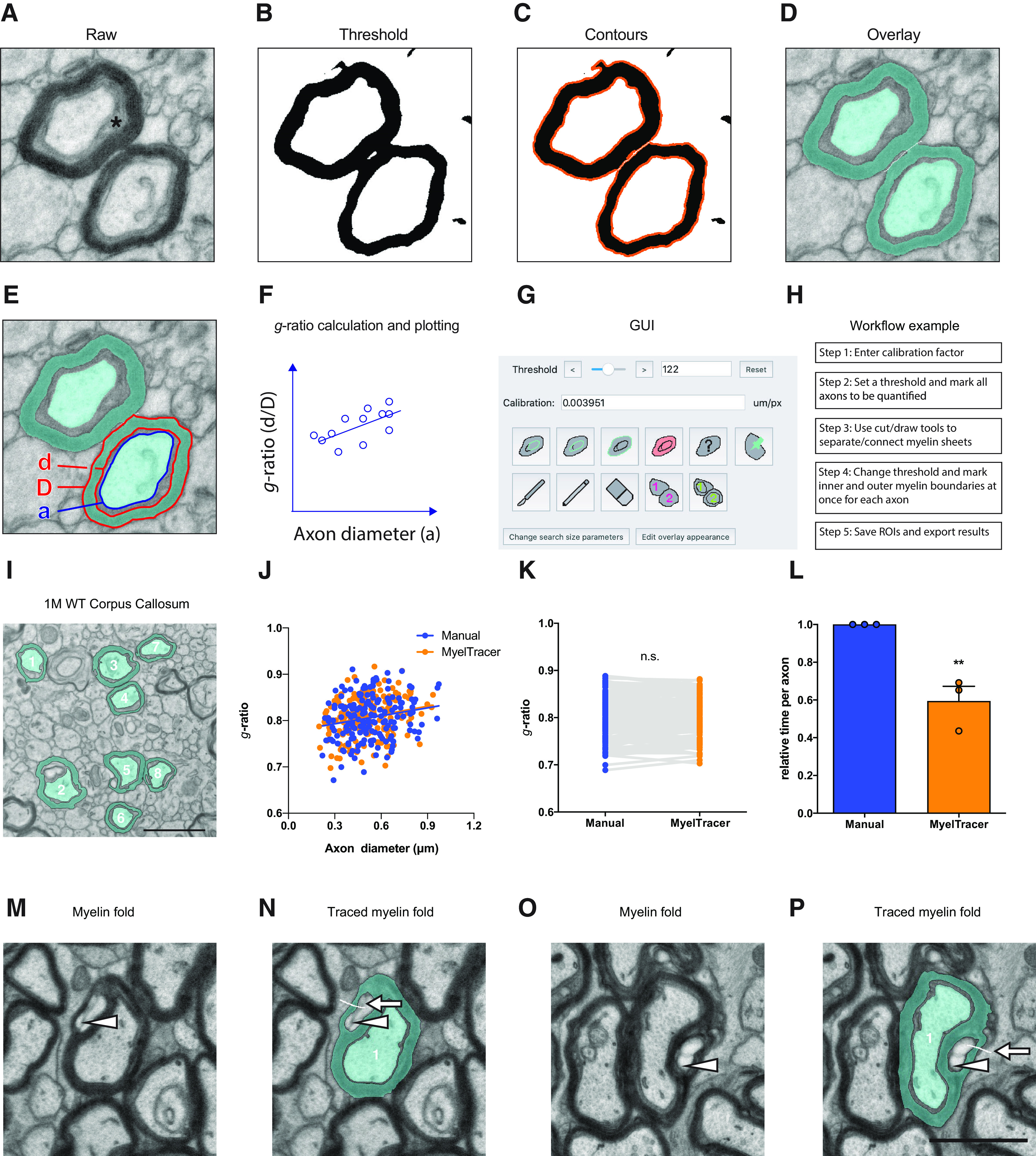
MyelTracer semi-automatically traces axons and myelin and returns results consistent with manual tracing. ***A–D***, Electron micrograph of dorsal corpus callosum, software-generated threshold overlays, and postselection overlay (blue) show different steps of the image analysis. * periaxonal space. ***E***, Electron micrograph with MyelTracer overlay and outlines for features including axon diameter (a), inner myelin diameter (d), and outer myelin diameter (D), each computed from the respective areas. ***F***, Schematic for *g*-ratio scatterplot illustrating how MyelTracer calculates the *g*-ratio. ***G***, Image of the main user interface of MyelTracer. ***H***, Schematic showing an example workflow using MyelTracer. ***I***, Representative electron micrograph of corpus callosum from one-month-old wild-type mice. ***J***, G-ratio scatter plot from manual and MyelTracer quantifications for the same axons. ANCOVA test *p* = 0.8996 (slopes) and *p* = 0.7044 (intercepts), *n* = 174 axons. ***K***, G-ratio from manual and MyelTracer quantification paired for the same axons. Student’s *t* test *p* = 0.9045, *n* = 174 axons. ***L***, Comparison of time consumption between manual quantification and quantification using MyelTracer. Individual data points represent the average time spent per axon per image; *p* = 0.0069; Student’s *t* test. Scale bar: 1 μm. ***M–P***, Illustration of MyelTracer’s ability to quantify myelin sheets with folds (arrowheads). Folds require the user to draw a white line (arrows) with the cut tool to connect the area inside the fold with the outside area for semi-automated detection. Scale bar: 1 μm. ns, no significance. **p < 0.01.

G-ratio quantification requires measuring the cross-sectional areas of axons and myelin sheaths, which is typically performed via manual tracing. Done manually, this process can be immensely time-consuming and laborious. To facilitate *g*-ratio quantification, we created MyelTracer by wrapping the powerful Python libraries provided by OpenCV in an easy-to-use GUI using PyQt5. MyelTracer performs automatic detection of contours using dynamic thresholding and separation of adjacent myelin sheaths based on user input. We packaged MyelTracer as a readily installable software for Windows and Mac OS X operating systems. Conceptually, MyelTracer takes a raw electron micrograph input image (Fig. 1*A*), converts it to grayscale, applies a bilateral filter, and thresholds it to a black and white binary image (Fig. 1*B*). MyelTracer extracts boundaries between the black and white pixels on the binary image and creates a contour image based on user-defined filtering parameters (Fig. 1*C*). For *g*-ratio plotting, MyelTracer is configured to measure the inner and outer myelin sheath as well as the axon diameter to account for periaxonal space present in electron micrographs (Fig. 1*A*). Using the GUI (Fig. 1*G*), the user can select the contours of axon, inner myelin, and outer myelin while adjusting the threshold to closely match the displayed contours to the underlying myelin ultrastructure. Once selected, MyelTracer groups the corresponding axons, inner myelin, and outer myelin together and overlays those selections on top of the original image (Fig. 1*D*). The software automatically computes diameters of perfect circles from area measurements and derives *g*-ratios by dividing inner myelin diameters by outer myelin diameters, which can then be plotted against the axon diameter (Fig. 1*E*,*F*).

To use MyelTracer efficiently, we recommend the following workflow (Fig. 1*H*, Extended Data 2). First, select all axons in the image by adjusting the threshold parameter to match contours to the axon shape. Second, adjust the threshold to match myelin sheaths. Third, check for contiguous contours of adjacent myelin sheaths as well as discontinuous contours around myelin sheaths. If necessary, use the cut tool to separate adjacent myelin sheaths, or use the draw tool to connect discontinuous myelin sheaths. Fourth, select the inner and outer myelin sheath contours, adjusting the threshold parameter to fit. Fifth, save the progress and export the result, which includes *g*-ratio measurements and an overlay (Fig. 1*D*). MyelTracer also provides a manual tracing option for otherwise untraceable features and a counting tool to calculate the percentage of myelinated axons. Together, these functions make MyelTracer an all-in-one software suite for studying myelin ultrastructure.

10.1523/ENEURO.0558-20.2021.ed2 Extended Data 2 Extended data 2 contains a Users’ Manual for MyelTracer and some illustrative images regarding micrograph quality. Download Extended Data 2, DOCX file.

### MyelTracer saves quantification time while maintaining accuracy

Myelin *g*-ratio quantification is routinely done by manually tracing the myelin sheath. Manual tracing is very time consuming but considered the standard practice for accurate results. To compare the accuracy of MyelTracer to manual tracing in moderately myelinated tissues, we measured *g*-ratios using both methods in the same electron micrographs of corpus callosum from one-month-old mice (Fig. 1*I*). Plotting the *g*-ratio as a function of axonal diameter, we did not find a significant difference between MyelTracer and manual quantification results (Fig. 1*J*, MyelTracer: slope 0.061, y-intercept 0.77, *n* = 173, manual: slope 0.057, y-intercept 0.77, *n* = 173; slopes *p* = 0.8996, intercept *p* = 0.7044^a^) and their averages (Fig. 1*K*, MyelTracer mean ± SD: 0.8072 ± 0.04417, *n* = 173, manual: 0.8066 ± 0.04220, *p* = 0.9045^b^). Next, to examine the amount of quantification time MyelTracer saves, the average amount of quantification time spent per axon was compared between these two methods. Using MyelTracer, significantly less time was spent per axon compared with manual tracing (Fig. 1*L*, MyelTracer mean: 0.593, manual: mean 1.0, *n* = 173 axons, three images, *p* = 0.0069^c^). Further, to exemplify that MyelTracer can trace myelin sheets containing folds, several such sheets were analyzed. Taking advantage of the cut tool, MyelTracer readily detects myelin folds and correctly quantifies the myelin sheet without including the area in the fold (Fig. 1*M–P*). Together, these data show that MyelTracer maintains a high level of accuracy, comparable to manual tracing, while saving ∼40% of time for the user.

**Figure 2.**
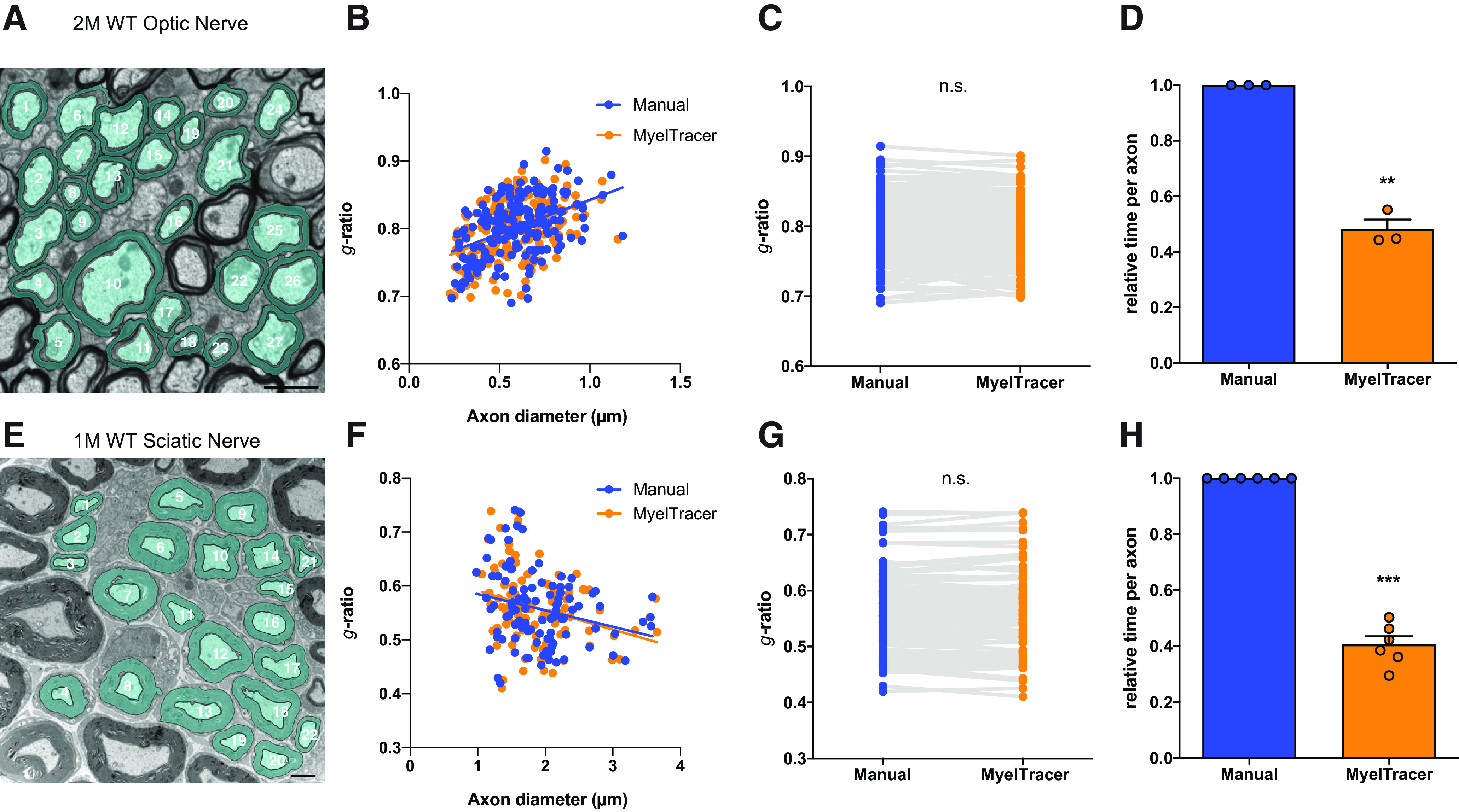
MyelTracer accurately returns *g*-ratios for myelinated tissues of varying axonal density. ***A***, Representative electron micrograph of the optic nerve at two months of age (2 M) with MyelTracer-generated overlays (blue). Scale bar: 1 μm. ***B***, *g*-Ratio scatter plot from manual and MyelTracer quantifications for the same axons. ANCOVA test *p* = 0.8311 (slopes) and *p* = 0.4695 (intercepts), *n* = 193 axons. ***C***, *g*-Ratios from manual and MyelTracer quantifications paired for the same axons. *p* = 0.3406, *n* = 193 axons. ***D***, Comparison of time consumption between manual quantification and quantification using MyelTracer. Individual data points represent the average time spent per axon per image; *p* = 0.0046. ***E***, Representative electron micrograph of the sciatic nerve at one month of age with MyelTracer-generated overlays (blue). Scale bar: 2 μm. ***F***, *g*-Ratio scatter plot from manual and MyelTracer quantifications for the same axons. ANCOVA test *p* = 0.7437 (slopes) and *p* = 0.8997 (intercepts), *n* = 104 axons. ***G***, *g*-Ratios from manual and MyelTracer quantifications paired for the same axons. *p* = 0.9146, *n* = 104 axons. ***H***, Comparison of time consumption between manual quantification and quantification using MyelTracer. Individual data points represent the average time spent per axon per image; *p* < 0.0001; Student’s *t* test. ns, no significance. **p < 0.01, ***p < 0.001.

Myelination is spatiotemporally regulated (Mayoral and Chan, 2016; Sun et al., 2018). As a result, there are variations in myelin sheath thickness and spatial density of myelinated axons across different types of tissues and developmental time points. To assess the feasibility of using MyelTracer for different types of tissue samples, electron micrographs from optic nerves and sciatic nerves were examined (Fig. 2*A*,*E*). In the optic nerve of two-month-old mice, plotting *g*-ratio as a function of axon diameter revealed no significant difference between the results from using MyelTracer and those from manual quantification (Fig. 2*B*, MyelTracer: slope 0.1046, y-intercept 0.7372, *n* = 193, manual: slope 0.1001, y-intercept 0.7427, *n* = 193, slopes *p* = 0.8311, intercept *p* = 0.4695^d^). We did not find a significant difference in *g*-ratio (Fig. 2*C*, MyelTracer mean ± SD: 0.7976 ± 0.04328, *n* = 193, manual: 0.8018 ± 0.04244, *p* = 0.3406^e^), but a significant reduction in time spent quantifying *g*-ratio (Fig. 2*D*, MyelTracer mean: 0.4808, manual: mean 1.0, *n* = 193 axons, three images, *p* = 0.0046^f^). In the sciatic nerve of one-month-old mice, regression relationships between *g*-ratio and axon diameter were not significantly different between using MyelTracer and manual quantification (Fig. 2*F*, MyelTracer: slope −0.0386, y-intercept 0.627, *n* = 104, manual: slope −0.030, y-intercept 0.615, *n* = 104, slopes *p* = 0.7437, intercept *p* = 0.8997^g^). Comparing the results from using MyelTracer and those from manual quantification in the sciatic nerve, there was no difference in *g*-ratio (Fig. 2*G*, MyelTracer mean ± SD: 0.5586 ± 0.07121, *n* = 104, manual: 0.5576 ± 0.06833, *p* = 0.9146^h^), while there was a significant reduction in time spent quantifying *g*-ratio (Fig. 2*H*, MyelTracer mean 0.4054, manual mean 1, *n* = 104 axons, 6 images, *p* < 0.0001^i^). Together, these findings demonstrate that MyelTracer saves 40–60% user quantification time and offers accuracy levels that are comparable to manual quantification.

### Myelin thickness abnormalities can be detected using MyelTracer

**Figure 3.**
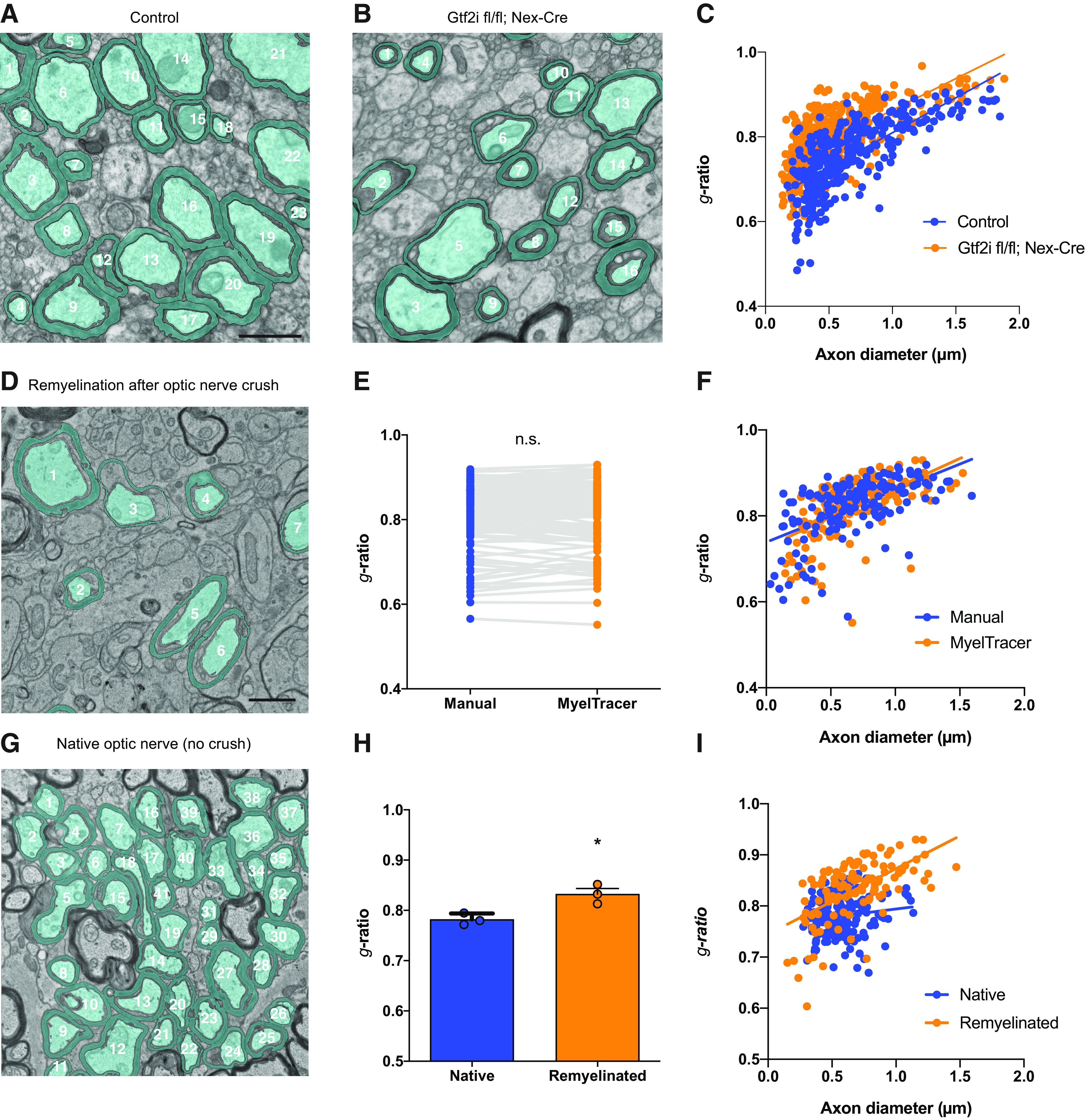
MyelTracer can be used to detect a myelin thickness abnormality in a Williams syndrome mouse model and to measure *g*-ratios in remyelination. ***A***, ***B***, Representative electron micrographs of the corpus callosum in control and Gtf2i fl/fl; Nex-Cre mice at one month of age with MyelTracer-generated overlays (blue). Scale bar: 1 μm. ***C***, G-ratio scatter plot from using MyelTracer for control and Gtf2i fl/fl; Nex-Cre mice; *p* < 0.0001; ANCOVA test; *n* = 3 mice, 332 axons for control and *n* = 3 mice, 455 axons for Gtf2i fl/fl; Nex-Cre mice. ***D***, Representative electron micrograph of the optic nerve 28 d after optic nerve crush with MyelTracer-generated overlays (blue). Scale bar: 1 μm. ***E***, G-ratio from manual and MyelTracer quantifications paired for the same axons. *p* = 0.9699, *n* = 155 axons. ***F***, G-ratio scatter plot from manual and MyelTracer quantifications for the same axons. ANCOVA test *p* = 0.1511 (slopes) and *p* = 0.8686 (intercepts), *n* = 155 axons. ***G***, Representative electron micrograph of the native control optic nerve with MyelTracer-generated overlays (blue). ***H***, G-ratio group comparison of axons from native control and remyelinated axons from mice following optic nerve crush; *p* < 0.0183; *t* test; *n* = 3 mice per group. ***I***, G-ratio scatter plot of axons from native control and remyelinated axons from mice following optic nerve crush. ANCOVA test *p* < 0.0001 (slopes), native control *n* = 183 axons, remyelinated *n* = 116 axons. ns, no significance. *p < 0.05.

Using MyelTracer for *g*-ratio quantification saves a significant amount of time and produces accurate results across different types of tissues from wild-type mice (Figs. 1, 2). In research settings, however, most investigators seek a tool to detect differences between genotypes or treatment paradigms. To test whether MyelTracer is suitable for detecting differences between two experimental groups, its performance was tested using electron micrographs from control (Gtf2i^fl/fl^, Nex-Cre−/−) and Gtf2i^fl/fl^; Nex-Cre^+/−^ mice, a model for Williams syndrome with a known hypomyelination phenotype (Barak et al., 2019). With their genotypes blinded, electron micrographs of corpus callosum from both groups at one month of age were analyzed using MyelTracer (Fig. 3*A*,*B*). Regression relationships between *g*-ratio and axon diameter were significantly different between control and Gtf2i^fl/fl^; Nex-Cre^+/−^ mice (Fig. 3*C*, control: slope 0.1649, y-intercept 0.6447, *n* = 332 axons, *n* = 3 mice, Gtf2i^fl/fl^; Nex-Cre^+/−^: slope 0.1487, y-intercept 0.7132, *n* = 787, *n* = 3 mice, slopes *p* = 0.1484, intercept *p* < 0.001^j^). The *g*-ratio measurements were higher in Gtf2i^fl/fl^; Nex-Cre^+/−^ mice than those in control mice, indicating a reduction in myelin sheath thickness, a result consistent with the original report (Barak et al., 2019). Together, these data demonstrate that MyelTracer is suitable for detecting differences in myelin *g*-ratios across different genotypes.

### MyelTracer can be used to quantify *g*-ratios in remyelination

MyelTracer readily recognizes well-developed myelin sheaths in micrographs of both central and peripheral nerves and axon tracts. In contrast to the relatively thick and compact myelin in these tissues, myelin in remyelinating tissues after injury is characterized by a thinner morphology (Franklin and ffrench-Constant, 2008; Duncan et al., 2017). To test MyelTracer’s ability to quantify *g*-ratios in tissues undergoing remyelination, we analyzed micrographs from an optic nerve crush model 28 d after the injury (Fig. 3*D*). Comparing the results from MyelTracer and those from manual quantification in this model, there was no difference in *g*-ratio (Fig. 3*E*, MyelTracer mean ± SD: 0.8212 ± 0.06843, *n* = 155, manual: 0.8210 ± 0.06944, *p* = 0.9699^k^). Further, regression relationships between *g*-ratio and axon diameter were not significantly different between MyelTracer and manual quantification (Fig. 3*F*, MyelTracer: slope 0.1484, y-intercept 0.7150, *n* = 155, manual: slope 0.1282, y-intercept 0.7273, *n* = 155, slopes *p* = 0.1511, intercept *p* = 0.8686^l^). To further test whether MyelTracer is suitable to detect the thinned myelin sheets in remyelination compared with native myelin sheets, its performance was tested using region-matched electron micrographs from remyelinated axons and axons from matched native control animals (Fig. 3*G*). Comparison of the average *g*-ratio per animal revealed significantly increased *g*-ratios in the remyelinated group compared with the native control group (Fig. 3*H*, native control mean ± SD: 0.7821 ± 0.01162, *n* = 3 mice, remyelinated: 0.8324 ± 0.01942, *n* = 3 mice, *p* = 0.0183^m^). Regression relationships between *g*-ratio and axon diameter were significantly different between the optic nerve axons in native control and remyelinated mice (Fig. 3*I*, native control: slope 0.03137, y-intercept 0.7627, *n* = 183 axons, remyelinated: slope 0.1281, y-intercept 0.7449, *n* = 116, *n* = 3 mice, slopes *p* < 0.0001^n^). Together, these results indicate that MyelTracer is suitable for *g*-ratio measurements in remyelination.

## Discussion

In this study, we show that MyelTracer can address the need for an easy-to-use, stand-alone software for streamlining *g*-ratio quantification. OpenCV, PyQt5, and fbs were used in Python to develop and package MyelTracer, an easy to install software with an intuitive GUI. MyelTracer utilizes contrast in electron micrographs to generate contours around axons and myelin sheaths. The user can select contours outlining myelin features of interest (axons, inner myelin, and outer myelin), which then allow MyelTracer to group corresponding features, measure their areas and calculate *g*-ratios. As an all-in-one platform for myelin analyses, MyelTracer can also be used to calculate the percentage of myelinated axons. Using MyelTracer, user quantification time is reduced by 40–60% depending on the region, and its accuracy is comparable to manual tracing. Using MyelTracer, a known hypomyelination phenotype of the Williams syndrome mouse model can be detected, confirming its suitability for studies assessing phenotypic changes in myelin sheaths. MyelTracer is freely available at https://github.com/HarrisonAllen/MyelTracer.

Myelin ultrastructure is highly heterogeneous with a wide range of axon calibers and myelin sheath morphologies, presenting a challenge for automated analysis. MyelTracer uses OpenCV to automatically contour cross-sections of axons and myelin sheaths in electron micrographs (Fig. 1*A–C*). The easy-to-use GUI allows users to intuitively interact with and fine-tune the results generated by the underlying computer vision algorithms (Fig. 1*D*,*G*). MyelTracer decreases analysis time by dramatically accelerating axon and myelin sheath contouring compared with manual tracing. This automated feature differentiates MyelTracer from existing non-automated tools, such as GRatio for ImageJ, which is an ImageJ plugin that facilitates grouping of traced structures and data export (Goebbels et al., 2010).

In addition to MyelTracer, several semi-automated and automated tools are available that exhibit different strengths and weaknesses. *AxonSeg* and *AxonDeepSeg* are MATLAB-based segmentation algorithms that are fully automated, enabling high-throughput measurements (Zaimi et al., 2016, 2018). These tools are useful for processing large datasets but require image preprocessing, adjustment of parameters in MATLAB, or additional training of the neural network using manually labeled images from user-specific datasets, which can be hurdles for implementation. Janjic et al. (2019) developed a high-fidelity tool using deep neural networks, which suits researchers with a computer science background seeking an advanced *g*-ratio quantification tool. While there are other useful tools available, MyelTracer’s intuitive GUI makes it a powerful tool allowing researchers to quantify myelin without computer science knowledge.

G-ratio quantification using MyelTracer was demonstrated to be accurate across different tissues, such as corpus callosum, optic nerve, and sciatic nerve (Figs. 1, 2). For these types of samples, there was a 40–60% reduction in time spent on quantification (Figs. 1*L*, 2*D*,*H*). The variation in the amount of time-reduction depended on the type of tissue. The smallest time-reduction was for corpus callosum of one-month-old mice. This is likely because of the heterogeneity in axonal caliber, axonal density, and the contrast level among the axon, periaxonal space, inner tongue, and the myelin sheath (Fig. 1*A*,*I*). In contrast, the greatest time-reduction was observed for sciatic nerve, which has clearly defined myelinated axons with homogeneous morphology, minimal periaxonal space and minimal myelin contiguity between adjacent axons (Fig. 2*E*,*H*). Comparing MyelTracer workflows for corpus callosum versus sciatic nerve, a major difference was that the latter required less manual input to separate contiguous myelin sheaths (step 3; Fig. 1*H*). Similar to manual tracing and other computational toolboxes, MyelTracer also relies on the quality of input images and therefore shows less effective performance for images with poor contrast levels, artifacts from inadequate perfusion or tissue handling.

The analysis of electron micrographs from Williams syndrome mice shows that MyelTracer readily detects abnormalities in myelination (Fig. 3*A–C*), demonstrating the suitability of MyelTracer for detecting differences in myelin *g*-ratio across different genotypes or treatment groups. In such studies, MyelTracer will decrease user quantification time, and streamline data generation for publication purposes.

Extending the application for MyelTracer, analysis of remyelinating axons 28 d after optic nerve crush shows that the software is suitable for *g*-ratio measurements in studies of remyelination (Fig. 3*D–I*). Myelin sheaths in remyelinating tissues are generally found to be thinner (Franklin and ffrench-Constant, 2008; Duncan et al., 2017), and they can appear almost discontinuous in a given sectioning plane. This presents a challenge to automation and may require user input using MyelTracer’s *Cut* and *Draw* tools as extensively outlined in the Users’ Manual (Extended Data 2). Notwithstanding these challenges, MyelTracer is suitable for the semi-automated tracing of micrographs in studies of remyelination provided that the myelin sheaths and axons display good contrast.

As a user-friendly all-in-one software suite, MyelTracer will also allow researchers to quantify the percentage of myelinated axons, which together with *g*-ratio, are the most commonly reported metrics in myelin studies. Beyond existing tools and MyelTracer, advances in computer vision and artificial intelligence may further advance data analysis methods and produce more powerful toolboxes to streamline quantitative analysis of myelin ultrastructure.

## Synthesis

Reviewing Editor: Isabel Perez-Otaño, Instituto de Neurociencias Decisions are customarily a result of the Reviewing Editor and the peer reviewers coming together and discussing their recommendations until a consensus is reached. When revisions are invited, a fact-based synthesis statement explaining their decision and outlining what is needed to prepare a revision will be listed below. The following reviewer(s) agreed to reveal their identity: Annik Barron.

Both reviewers found value in your paper, and considered MyelTracer a welcome addition to the world of myelin quantification. Nevertheless, both agreed that validation of the utility of the software to quantify remyelination is needed. In addition the challenges posed to automated myelin sheath measures by presence of myelin folds as seen in dysmyelination needs to be addressed. They would also like to see that the software works for routine micrographs, and better definitions of g-ratios taking into account parameters such as the inner tongue. Also, I´d suggest to remove developmental myelination from the abstract as developmental validation at different stages was not conducted

Reviewer comments

Reviewer#1:

This paper is of interest to the glial community focusing on myelin and de-myelination remyelination. The field is forced to quantify the number of myelinating or re-myelinating axons or highlight quantitatively developmental or adult anomalies or the efficacy of repair with or without a pharmacological or cell therapy approach. While the developmental aspect has been well integrated (3 different CNS tissues and WT and Hypomyelinated mice), the repair aspect has been neglected and would optimize the paper impact. Remyelination is characterized by thin myelin sheath, and I wonder whether the system would be sensitive enough to capture this item.

It is also crucial when dealing with mice to have the appropriate number of animals for statistic analysis. I would advise to outline clearly from the start, the type of mice that have been used as well as their number per group (method section) : how many of them were prepared and imaged?.

Finally, it would be appreciated to have a proper explantation on the use of the software.

There are a few minor remarks:

pages should be outnumber to facilitate comments

References should be updated. 1st {section sign} introduction: there are more recent reviews on CNS and PNS myelin than Simons and Trotter 2007; Sherman and Brophy are not in the list

A ref should be added after “Myelination is spatiotemporally regulated” (2nd paragaph of results).

Reviewer#2

I couldn’t download the software to test it, so it’s difficult for me to test its ease-of-use. The figures presented look impressive, with good discrimination of myelin sheath, inner tongue and axon and evidence of reproducibility and time-saving. As such, I think this is something that could be made available. I have however some comments that should be addressed prior to any publication

The realisation that the inner tongue can change quite significantly in thickness e.g. in myelin plasticity has rather complicated the use of g ratio analysis. There are still descriptions of the g ratio that make the assumption that the inner surface of the myelin sheath and the outer surface of the axon are in direct opposition, and measure accordingly. The authors should be clear, and illustrate, exactly what they are measuring to calculate the g ratio. Also, they should stress that a potential further value of this software tool is that it allows measurement of the inner tongue volume which will be of great value in future work examining myelin plasticity and other areas in which the sheath changes shape.

The distinction between sheath, inner tongue and axon is clearly essential, but it’s not clear just how easy this is on a range of electron micrographs. Its easy on the ones shown, but given the normal variation in fixation, staining et cetera seen with EM it would be useful to know what proportion of routinely obtained micrographs were usable?

It’s noticeable that none of the sheaths shown have folds and/or loops of myelin separated from the axon. These are in fact quite common, especially in experimental and pathological situations. It’s essential that the tool can handle these without affecting its accuracy. This needs to be described and illustrated

A very common use will be assessing remyelination and measuring the thin myelin sheaths that result. It’s important that the authors include an analysis of remyelination in this paper to show that the very thin sheaths can be measured.

## Author Response

Detailed response to reviewers’ comments:

We thank the reviewers for their support of this work and their constructive comments. As detailed below, we have revised the manuscript according to reviewers’ comments by including additional analysis on remyelination and additional information. We believe that the manuscript is significantly improved, and we thank the reviewers for their help.

Reviewer 1

Major comments

1.This paper is of interest to the glial community focusing on myelin and de-myelination remyelination. The field is forced to quantify the number of myelinating or re-myelinating axons or highlight quantitatively developmental or adult anomalies or the efficacy of repair with or without a pharmacological or cell therapy approach. While the developmental aspect has been well integrated (3 different CNS tissues and WT and Hypomyelinated mice), the repair aspect has been neglected and would optimize the paper impact. Remyelination is characterized by thin myelin sheath, and I wonder whether the system would be sensitive enough to capture this item.

Authors’ reply:

Thank you for raising the question of the software’s ability to measure g-ratios in remyelinating tissues. We agree that this would maximize the impact of the paper and tested it accordingly. To benchmark the software’s performance in remyelinating tissues, we chose an optic nerve crush model and incorporated the results in Figure 3D-F. We find that the software can accurately measure the thin myelin sheaths in the remyelinating optic nerve. We also included a corresponding paragraph in the discussion.

Lines 242-254 in results now read:”MyelTracer readily recognizes well-developed myelin sheaths in micrographs of both central and peripheral nerves and axon tracts. In contrast to the relatively thick and compact myelin in these tissues, myelin in remyelinating tissues after injury is characterized by a thinner morphology (Franklin and ffrench-Constant, 2008; Duncan et al., 2017). To test MyelTracer’s ability to quantify g-ratios in tissues undergoing remyelination, we analyzed micrographs from an optic nerve crush model 28 days after the injury (Figure 3D). Comparing the results from MyelTracer and those from manual quantification in this model, there was no difference in g-ratio (Figure 3E, MyelTracer mean {plus minus} SD: 0.8212 {plus minus} 0.06843, n = 155, Manual: 0.8210 {plus minus} 0.06944, p = 0.9699k). Further, regression relationships between g-ratio and axon diameter were not significantly different between MyelTracer and manual quantification (Figure 3F, MyelTracer: slope 0.1484, y-intercept 0.7150, n = 155, Manual: slope 0.1282, y-intercept 0.7273, n = 155, slopes p = 0.1511, intercept p = 0.8686k). Together, these results indicate that MyelTracer is suitable for g-ratio measurements in remyelination.”

Lines 309-317 in the discussion now read:”Extending the application for MyelTracer, analysis of remyelinating axons 28 days after optic nerve crush shows that the software is suitable for g-ratio measurements in studies of remyelination (Figure 3D-F). Myelin sheaths in remyelinating tissues are generally found to be thinner (Franklin and ffrench-Constant, 2008; Duncan et al., 2017), and they can appear almost discontinuous in a given sectioning plane. This presents a challenge to automation and may require user input using MyelTracer’s Cut and Draw tools as extensively outlined in the Users’ Manual (Extended Data 2). Notwithstanding these challenges, MyelTracer is suitable for the semi-automated tracing of micrographs in studies of remyelination provided that the myelin sheaths and axons display good contrast.”

2.It is also crucial when dealing with mice to have the appropriate number of animals for statistical analysis. I would advise to outline clearly from the start, the type of mice that have been used as well as their number per group (method section) : how many of them were prepared and imaged?

Authors’ reply:

We thank the reviewer for pointing out that this important piece of information was incomplete. We have revised both the methods section and figure captions to reflect the animal and axon numbers clearly.

Lines 143-148: “For each experiment comparing manual tracing with MyelTracer, several micrographs were obtained from preparations of two to three mice per anatomic region. Within these micrographs, at least 100-300 axons were analyzed depending on the anatomical region. For testing MyelTracer’s capability to detect a hypomyelination phenotype in a Williams Syndrome model, several micrographs from three mice per genotype with a total of 332 and 455 axons were analyzed for control and Gtf2i fl/fl; Nex-Cre mice, respectively"

Lines 333-334, 346-347, 352-353, 366, 369-371: all figure captions were updated to reflect axon numbers.

3.Finally, it would be appreciated to have a proper explanation on the use of the software.

Authors’ reply:

We thank the reviewer for pointing out the need for a more thorough explanation on the use of the software. We have incorporated an instruction manual for the software as Extended Data 2.

Reviewer 2

Major comments

1.The realisation that the inner tongue can change quite significantly in thickness e.g. in myelin plasticity has rather complicated the use of g ratio analysis. There are still descriptions of the g ratio that make the assumption that the inner surface of the myelin sheath and the outer surface of the axon are in direct opposition, and measure accordingly. The authors should be clear, and illustrate, exactly what they are measuring to calculate the g ratio. Also, they should stress that a potential further value of this software tool is that it allows measurement of the inner tongue volume which will be of great value in future work examining myelin plasticity and other areas in which the sheath changes shape.

Authors’ reply:

We appreciate this important note and suggestion to measure the myelin tongue. In response, we first added a diagram about how g-ratio is calculated in the software in Figure 1E-F illustrating that the software does not assume that the inner surface of the sheath and the outer surface of the axon are in direct apposition. Secondly, to explore the possibility of measuring the inner tongue area, we incorporated a free measurement tool in the software. This tool can be customized to measure any area/region a researcher interested in, including but not limit to mitochondria and inner tongue. As an alternative, we contemplated including an automated output of the inner tongue area as the difference between the outer surface of the axon and the inner sheath of the myelin. However, since this space does not always equal the inner tongue, we ultimately decided to leave this calculation to the user.

2.The distinction between sheath, inner tongue and axon is clearly essential, but it’s not clear just how easy this is on a range of electron micrographs. It’s easy on the ones shown, but given the normal variation in fixation, staining et cetera seen with EM it would be useful to know what proportion of routinely obtained micrographs were usable?

Authors’ reply:

We appreciate this point and agree that high quality micrographs are often required for accurate distinction of these features. This important point holds true for both manual measurements and quantification using MyelTracer. To make requirements clear, we added information regarding some basic requirement of micrograph quality. These include compact myelin sheath and good contrast between myelin sheath and surrounding. As a reference for the user, we also added example images to the users’ manual (Extended Data 2) that provide guidance for the required image quality.

3.It’s noticeable that none of the sheaths shown have folds and/or loops of myelin separated from the axon. These are in fact quite common, especially in experimental and pathological situations. It’s essential that the tool can handle these without affecting its accuracy. This needs to be described and illustrated

Authors’ reply:

We agree that it is essential that the software can handle such structures. MyelTracer can measure nearly all sheaths that can be measured manually, including those that contain loops and folds. To address this point, we included an example and instruction on how to measure loops of myelin separated from the axon in the user’s manual (Extended Data 2).

4.A very common use will be assessing remyelination and measuring the thin myelin sheaths that result. It’s important that the authors include an analysis of remyelination in this paper to show that the very thin sheaths can be measured.

Authors’ reply:

Thank you for raising the question of the software’s ability to measure g-ratios in remyelinating tissues. We agree that this would maximize the impact of the paper and tested it accordingly. To benchmark the software’s performance in remyelinating tissues, we chose an optic nerve crush model and incorporated the results in Figure 3D-F. We find that the software can accurately measure the thin myelin sheaths in the remyelinating optic nerve. We also included a corresponding paragraph in the discussion.

Lines 242-254 in results now read:

"MyelTracer readily recognizes well-developed myelin sheaths in micrographs of both central and peripheral nerves and axon tracts. In contrast to the relatively thick and compact myelin in these tissues, myelin in remyelinating tissues after injury is characterized by a thinner morphology (Franklin and ffrench-Constant, 2008; Duncan et al., 2017). To test MyelTracer’s ability to quantify g-ratios in tissues undergoing remyelination, we analyzed micrographs from an optic nerve crush model 28 days after the injury (Figure 3D). Comparing the results from MyelTracer and those from manual quantification in this model, there was no difference in g-ratio (Figure 3E, MyelTracer mean {plus minus} SD: 0.8212 {plus minus} 0.06843, n = 155, Manual: 0.8210 {plus minus} 0.06944, p = 0.9699k). Further, regression relationships between g-ratio and axon diameter were not significantly different between MyelTracer and manual quantification (Figure 3F, MyelTracer: slope 0.1484, y-intercept 0.7150, n = 155, Manual: slope 0.1282, y-intercept 0.7273, n = 155, slopes p = 0.1511, intercept p = 0.8686k). Together, these results indicate that MyelTracer is suitable for g-ratio measurements in remyelination.”

Lines 309-317 in the discussion now read:

"Extending the application for MyelTracer, analysis of remyelinating axons 28 days after optic nerve crush shows that the software is suitable for g-ratio measurements in studies of remyelination (Figure 3D-F). Myelin sheaths in remyelinating tissues are generally found to be thinner (Franklin and ffrench-Constant, 2008; Duncan et al., 2017), and they can appear almost discontinuous in a given sectioning plane. This presents a challenge to automation and may require user input using MyelTracer’s Cut and Draw tools as extensively outlined in the Users’ Manual (Extended Data 2). Notwithstanding these challenges, MyelTracer is suitable for the semi-automated tracing of micrographs in studies of remyelination provided that the myelin sheaths and axons display good contrast.”

Minor comments:

1.Pages should be outnumber to facilitate comments.

Authors’ reply: Thank you for pointing this out. We have now properly numbered the pages and lines.

2.References should be updated. 1st section of introduction: there are more recent reviews on CNS and PNS myelin than Simons and Trotter 2007; Sherman and Brophy are not in the list

Authors’ reply: Thank you very much for pointing this out and for your keen observation. We have now added more recent reviews and made sure Sherman and Brophy are properly included in the bibliography.

Lines 30-32 now read: “Oligodendrocytes and Schwann cells produce the myelin sheath through lamellar enwrapping of axons (Simons and Trotter, 2007; Salzer, 2015; Hughes and Appel, 2016; Stadelmann et al., 2019).”

More recent reviews added:

-Stadelmann C, Timmler S, Barrantes-Freer A, Simons M. Myelin in the central nervous system: structure, function, and pathology. Physiological reviews. 2019 Jul 1;99(3):1381-431.

-Hughes EG, Appel B. The cell biology of CNS myelination. Curr Opin Neurobiol. 2016;39:93-100. doi:10.1016/j.conb.2016.04.013

-Salzer JL. Schwann cell myelination. Cold Spring Harb Perspect Biol. 2015;7(8):a020529. Published 2015 Jun 8. doi:10.1101/cshperspect.a020529

3.A ref should be added after “Myelination is spatiotemporally regulated” (2nd paragraph of results).

Authors’ reply: Thank you for pointing out the lack of a reference here. We now added a recent article by Sun et al as well as an excellent review on the topic.

Lines 201-203 now read: “Myelination is spatiotemporally regulated (Mayoral and Chan, 2016; Sun et al., 2018). As a result, there are variations in myelin sheath thickness and spatial density of myelinated axons across different types of tissues and developmental time points.”

-Sun, L. O., Mulinyawe, S. B., Collins, H. Y., Ibrahim, A., Li, Q., Simon, D. J., ... & Barres, B. A. (2018). Spatiotemporal control of CNS myelination by oligodendrocyte programmed cell death through the TFEB-PUMA axis. Cell, 175(7), 1811-1826.

-Mayoral, Sonia R., and Jonah R. Chan. “The environment rules: spatiotemporal regulation of oligodendrocyte differentiation.” Current opinion in neurobiology 39 (2016): 47-52.
