# Supplementary material for: MyelTracer: A Semi-Automated Software for Myelin g-Ratio Quantification
Source: eNeuro. 2021 Jul 21;8(4):ENEURO.0558-20.2021. doi: 10.1523/ENEURO.0558-20.2021 (PMC8298095; doi:10.1523/ENEURO.0558-20.2021)
Supplement: Extended Data 2 — contains a Users’ Manual for MyelTracer and some illustrative images regarding micrograph quality. Download Extended Data 2, DOCX file. [file enu-eN-OTM-0558-20-s04.docx]

**MyelTracer: A semi-automated software for myelin *g*-ratio quantification**

**Users’ Manual**


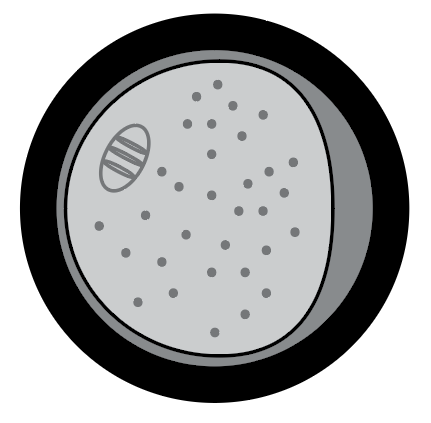


**Contents**

[Tools and settings of MyelTracer 2](#_Toc68868411)

[Workflow example 3](#_Toc68868412)

[Data examples 4](#_Toc68868413)

[Examples of micrographs that are easy to quantify using MyelTracer 4](#_Toc68868414)

[Examples of micrographs that are difficult to quantify using MyelTracer 4](#_Toc68868415)

[Troubleshooting 5](#_Toc68868416)

[Tips 5](#_Toc68868417)

# Tools and settings of MyelTracer

| **Tool** | **Explanation** | **Keyboard shortcut** |
| --- | --- | --- |
| 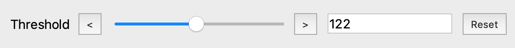 | **Threshold** changes the contouring and is the key parameter to change to align contours to axon and myelin features. | Arrow keys |
| 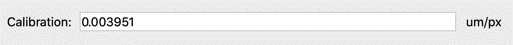 | **Calibration** sets the scale and ensures that axon diameter is returned correctly. | N/A |
| 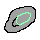 | **Axon tool** selects axons. | 1 |
| 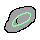 | **Inner myelin tool** selects the inner myelin sheath. | 2 |
| 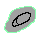 | **Outer myelin tool** selects the outer myelin sheath. | 3 |
| 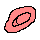 | **Deselection tool** removes current selection. | 4 |
| 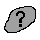 | **Information tool** indicates missing features on a given selection (e.g., a selection may be missing the inner myelin sheath). | 5 |
| 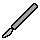 | **Cut tool** connects bright areas and serves to separate adjacent myelin sheaths or to sever off dark areas. | Q  *Shift + click for straight lines.* |
| 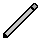 | **Draw tool** connects dark areas and serves to connect myelin sheaths interrupted by areas of low contrast. | W  *Shift + click for straight lines.* |
| 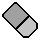 | **Eraser tool** removes unwanted lines or points. | E |
| 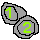 | **Myelinated counter** allows the user to mark myelinated axons. | T |
| 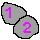 | **Unmyelinated counter** allows the user to mark unmyelinated axons. | R |
| 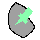 | **Misc selection tool** allows the user to count other features of interest. | 6 |
| 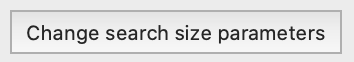 | **Change search size parameters** allows the user to adjust the *min* and *max* size of axons. | N/A |
| 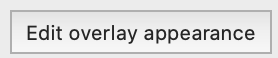 | **Edit overlay appearance** allows the user to adjust the transparency of overlays and outline thickness. | N/A |

# Workflow example

Different workflows are possible using MyelTracer, and each user may find a workflow that works best for them. Overall, we find that it is most efficient to work using MyelTracer keyboard shortcuts (as indicated above) as well as generic shortcuts (e.g., control + Z to undo or control + Z to redo, space + click to pan the image, control + to zoom in, control – to zoom out). Zoom scrolling can be deactivated in the settings, and assigned shortcuts can be seen in the dropdown menu as well. Axons and myelin selection tools also allow for manual tracing by *clicking + dragging*.

1. Open MyelTracer and load a micrograph using *File -> New*
2. Select image import quality that is supported by the computing power of your machine. We recommend using at least 70% to 80% of original quality.
3. Enter calibration factor (can be measured by ImageJ if unknown).
4. Set threshold so contouring will match axons. Mark all axons to be quantified while making finer adjustments in threshold and using the cut and draw tool as needed.
5. Use cut and draw tool to separate/connect myelin sheaths.
6. Change threshold and mark inner and outer myelin boundaries at once for each axon while using cut and draw tools as needed.
7. Save ROIs using *File -> Save As*
8. Export data using *File -> Export*
9. If desired, reopen and continue feature tracing using
   1. *File -> New* -> Image
   2. *File -> Open -> Data*. (Always use the same image quality for opening images as used for initial tracing and saving of the data).

# Data examples

MyelTracer operates based on differences in contrast. Hence, images with more compact myelin and better contrast require fewer manual inputs and are thus much easier to quantify. Below, we provide images to illustrate which micrographs are suitable for quantification using MyelTracer.

## Examples of micrographs that are easy to quantify using MyelTracer

| P30 Sciatic Nerve | P30 Corpus Callosum | P60 Optic Nerve |
| --- | --- | --- |
| 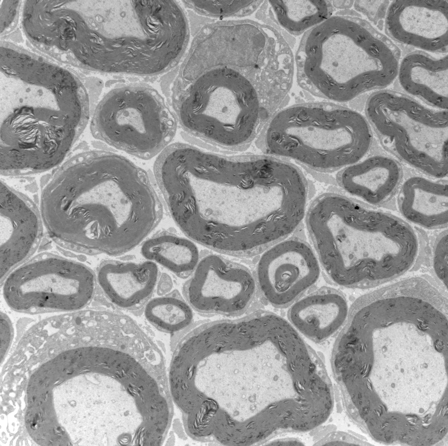 | 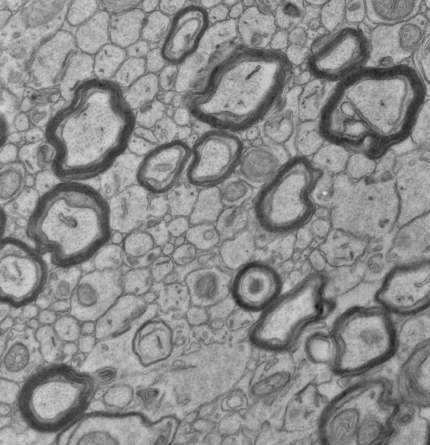 | 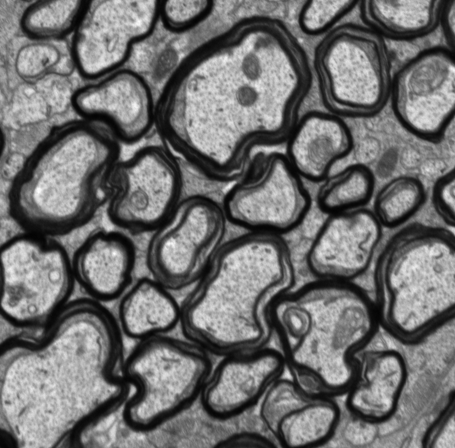 |

General features of micrographs that are well suitable for MyelTracer are relatively high contrast, largely continuous myelin sheaths, and spatially separated myelin sheaths.

## Examples of micrographs that are difficult to quantify using MyelTracer

| Highly dense areas | Low contrast images |
| --- | --- |
| 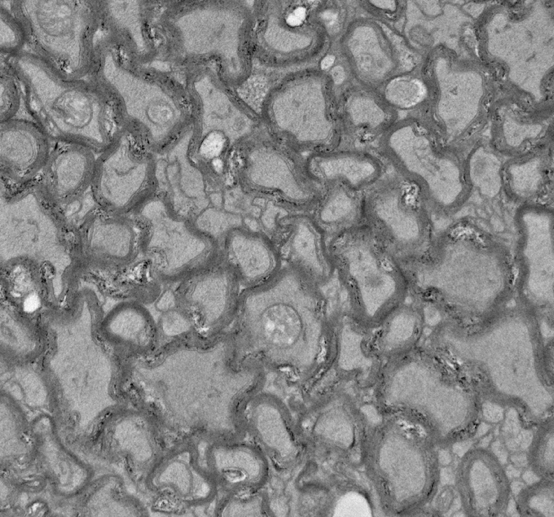 | 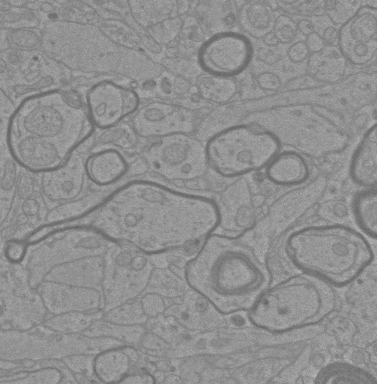 |

General features of micrographs which can be traced by MyelTracer but may preclude significant time savings include:

- relatively low contrast
- high proportion of discontinuous myelin sheaths
- high proportion of myelin sheaths in close apposition (“touching”)

# Troubleshooting

| **Issue** | **Solution** |
| --- | --- |
| Software does not open on Mac systems due to unidentified developer error. | Right-click on application and choose open and trust the application. |
| Contours are not forming around features (axon, inner myelin, outer myelin) | Use cut tool to sever off myelin from adjacent axons and draw tool to connect discontinuous portions of myelin.  Use draw tool to “bridge” areas of lower contrast in myelin. |
| Large axons are not being recognized | Change search size parameters. |
| Small axons are not being recognized | Change search size parameters. |
| The software lags | Load the image at lower resolution. |
| Saved mask does not match reopened image | Always choose same image quality as used for mask generation. |

# Tips

| 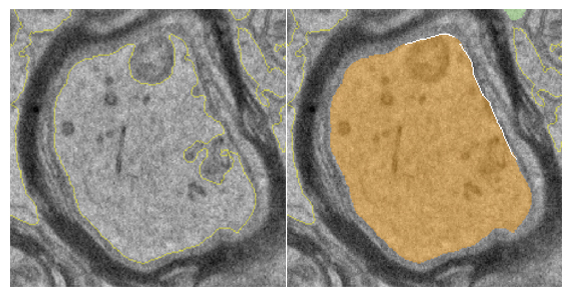 | Use the *Cut* tool to bridge the edge of axon (as shown by white line above). |
| --- | --- |
| 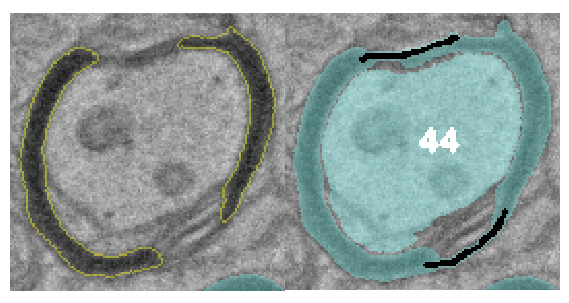 | Use the *Draw* tool to draw a single line helping MyelTracer to recognize discontinuous or relative low contrast myelin sheaths (as shown by black line above). |
| 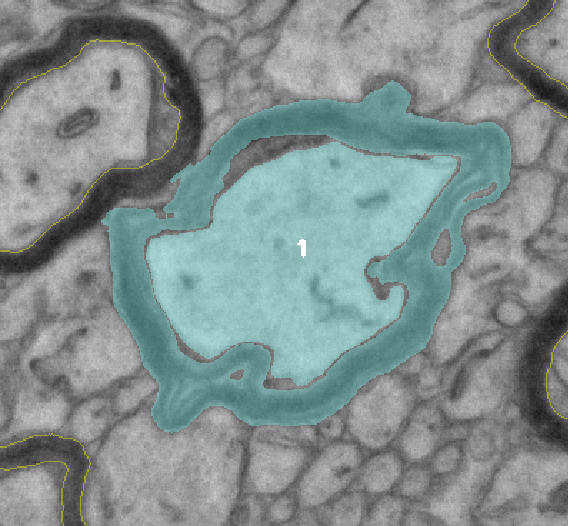 | Trace sheets with complex loops and folds. If needed, disconnect or connect with *Cut* and *Draw* tools. |
